# A multi-predator trophic database for the California Current Large Marine Ecosystem

**DOI:** 10.1038/s41597-023-02399-2

**Published:** 2023-07-27

**Authors:** Joseph J. Bizzarro, Lynn Dewitt, Brian K. Wells, K. Alexandra Curtis, Jarrod A. Santora, John C. Field

**Affiliations:** 1 Fisheries Collaborative Program, Cooperative Institute for Marine Ecosystems and Climate, University of California, Santa Cruz, 110 McAllister Way, Santa Cruz, California 95060 USA; 2 Fisheries Ecology Division, Southwest Fisheries Science Center, National Marine Fisheries Service, National Oceanic and Atmospheric Administration, 110 McAllister Way, Santa Cruz, California 95060 USA; 3 Environmental Research Division, Southwest Fisheries Science Center, National Marine Fisheries Service, National Oceanic and Atmospheric Administration, 110 McAllister Way, Santa Cruz, California 95060 USA; 4 Marine Mammal and Turtle Division, Southwest Fisheries Science Center, National Marine Fisheries Service, National Oceanic and Atmospheric Administration, 8901 La Jolla Shores Dr., La Jolla, California 92037 USA

**Keywords:** Food webs, Databases, Ecosystem ecology

## Abstract

The California Current Trophic Database (CCTD) was developed at NOAA Southwest Fisheries Science Center in collaboration with numerous diet data contributors. We compiled the CCTD from twenty-four data sets, representing both systematic collections and directed trophic studies. Diet composition data, including stomach and scat samples, were obtained from 105,694 individual predators among 143 taxa collected throughout the California Current Large Marine Ecosystem (CCLME) from 1967–2019. Predator taxa consist of squids (n = 5), elasmobranchs (n = 13), bony fishes (n = 118), and marine mammals (n = 7). Extensive time series are available for some predators (e.g., California Sea Lion, Pacific Hake, Chinook Salmon). The CCTD represents the largest compilation of raw trophic data within the CCLME, allowing for more refined analyses and modeling studies within this region. Our intention is to further augment and periodically update the dataset as additional historical or contemporary data become available to increase its utility and impact.

## Background & Summary

Quantifying trophic interactions, and associated spatiotemporal variability within such interactions, is fundamental to parameterizing ecosystem models and evaluating strategies for ecosystem-based fisheries management (EBFM). Quantitative diet composition data that document trophic relationships among species in marine communities can enable an improved understanding of ecosystem structure and dynamics^1^, trophic cascades^2^, and the identification of trade-offs and reference points^3^. In the California Current Large Marine Ecosystem (CCLME), recent population crises such as seabird and sea lion unusual mortality events, a salmon population collapse, and increasing whale entanglements, have raised the level of interest in the causes and consequences of climate-dependent shifts in trophic dynamics. Accurately predicting the effects of dynamic predator populations requires a comprehensive understanding of their trophic role within the ecosystem. For example, the success of protected species management has resulted in greater populations of marine mammals in the CCLME over the last 40 years^4,5^. This increase in predator abundance has altered population parameters such as natural mortality rates for some prey species of commercial or recreational importance, and presents competing objectives for fisheries and EBFM^6–8^. Balancing recovery of predators and prey populations requires an understanding of the underlying trophic interactions driving the ecosystem^9–11^.

Although summarized food habits information is adequate for some purposes, individual-based diet estimates are important to assess dietary variability (e.g., spatial, temporal, and ontogenetic) of sampled populations. Several studies summarize previously published food habits studies in the CCLME^12,13^, but they are limited to generalized information summarized from previously published studies which do not provide data at the resolution of an individual predator. Summarizing such information represents a major contribution, but as the value of food habits information continues to be recognized with respect to informing and parameterizing single species, multispecies, and ecosystem models, the ability for ecosystem modelers to access and incorporate raw data is invaluable. Individual-based food habits databases have been developed in other marine regions to support a suite of research and modeling efforts, including the Scotian Shelf^14^, Aleutian Islands, Gulf of Alaska, and eastern Bering Sea^15^, and throughout the North Pacific^16^, and provide users with access to raw data. Raw data provide a basis to better evaluate and quantify spatial and temporal patterns of variability within and among predator species and size classes, and among different studies and sampling regimes^17,18^.

To benefit scientists and fisheries management, we designed and developed a relational trophic database, termed the California Current Trophic Database (CCTD), consisting of a diverse array of marine predator taxa. Individual-based diet composition data, derived from stomach or scat samples, were located through an initial discovery period consisting of communications with potential and known data holders. Once contributed, we standardized the data so that information from disparate data sets could be effectively combined. The CCTD is a first step towards a comprehensive spatiotemporally explicit database for the CCLME. Our intention is to augment and periodically update the database as additional historical or contemporary data become available to increase its utility and impact.

## Methods

### Data collection and processing

Unlike most food habit databases for marine predators that are developed from standardized, regular or periodic fisheries surveys (e.g.^19,20^), the CCTD was compiled from a broad range of data sets, representing both systematic collections and data collected during directed trophic studies. Data contributors varied and included National Marine Fisheries Service (NMFS) scientists, graduate students, and academic researchers. The construction of a CCLME food habits database was prioritized by NOAA Southwest Fisheries Science Center (SWFSC), who initially compiled a list of potential data holders among staff and affiliates. Communication with this initial list of potential contributors led to a broader search of Northwest Fisheries Science Center and Alaska Fisheries Science Center personnel and unaffiliated researchers. We sought individual-based diet composition data for any marine predator in the greater CCLME. We also requested measurements of prey size and all temporal, spatial, and environmental data associated with stomach (for cephalopods, fishes, and cetaceans) or scat (for pinnipeds) samples. Individual-based food habits data were submitted directly from collaborators or were downloaded from published, web-based data sets as.csv,.xlsx, or.txt files. Any derivative publications and supporting information and materials describing methods of sample collection, processing, and identification were additionally obtained. In total, twenty-four data sets were contributed from collaborators (Table 1). Details regarding sample collection and processing are contained in published literature associated with each data set (Table 1). These details were not fully documented for one data and are therefore provided as Supplemental Methods.

**Table 1:** Data sources included in the California Current Trophic Database.

Data Set	Citation(s)	Predator Taxa	Prey Size	Empties/Blanks	O	N	W	V
1	^15,28,29^	Slope Groundfishes	x	x	x	x	x	x
2	^30^	Lingcod		x	x	x	x	
3	^27,31^	Pacific Hake		x	x	x		
4	^32,33^	Skates			x			x
5	^34^	Yellowtail Rockfish			x	x	x	
6	^35–39^	California Sea Lion	x	x	x	x	x	
7	^40–42^	Forage Fishes	x	x	x	x	x	x
8	^42^	Pelagic Fishes/Squid	x	x	x	x	x	x
9	^43^	Juvenile Salmon	x	x	x	x	x	
10	^44^	Pelagic Predators	x	x	x	x	x	
11	^45^	Albacore	x	x	x	x	x	
12	^46^	Bluefin Tuna	x	x	x	x		
13	^47,48^	Pink Salmon, Chum Salmon		x	x	x	x	
14	^49^	Chinook Salmon, Coho Salmon	x	x	x	x		x
15	^50,51^	Humboldt Squid	x	x	x	x		
16	^52^	Chinook Salmon	x	x	x	x		x
17	^53–55^	Salmon	x	x	x	x	x	
18	^56^	Northern California Demersal Fishes	x	x	x	x	x	x
19	^57^	Southern California Demersal Fishes	x	x	x	x	x	x
20	^56^, Supplemental Methods	Slope Groundfishes	x	x	x	x	x	x
21	^58^	Gopher Rockfish	x	x	x	x	x	x
22	^59^	Pinnipeds	x		x	x	x	x
23	^60^	Harbor Seal	x	x	x	x	x	x
24	^61–66^	Sharks/Dolphins/Swordfish	x	x	x	x	x	x

Citations are provided for publications that either described or leveraged each data set and are included in Literature Cited. Supplemental Methods are provided for one data set (20) because methods were not completely documented in the associated publication.  x = these types of data are included. Empties/Blanks = empty stomach samples or blank scat samples are included. Diet metrics = O (occurrence, N (number), W (weight), and V (volume).

### Data standardization and synthesis

**Fig. 1:**
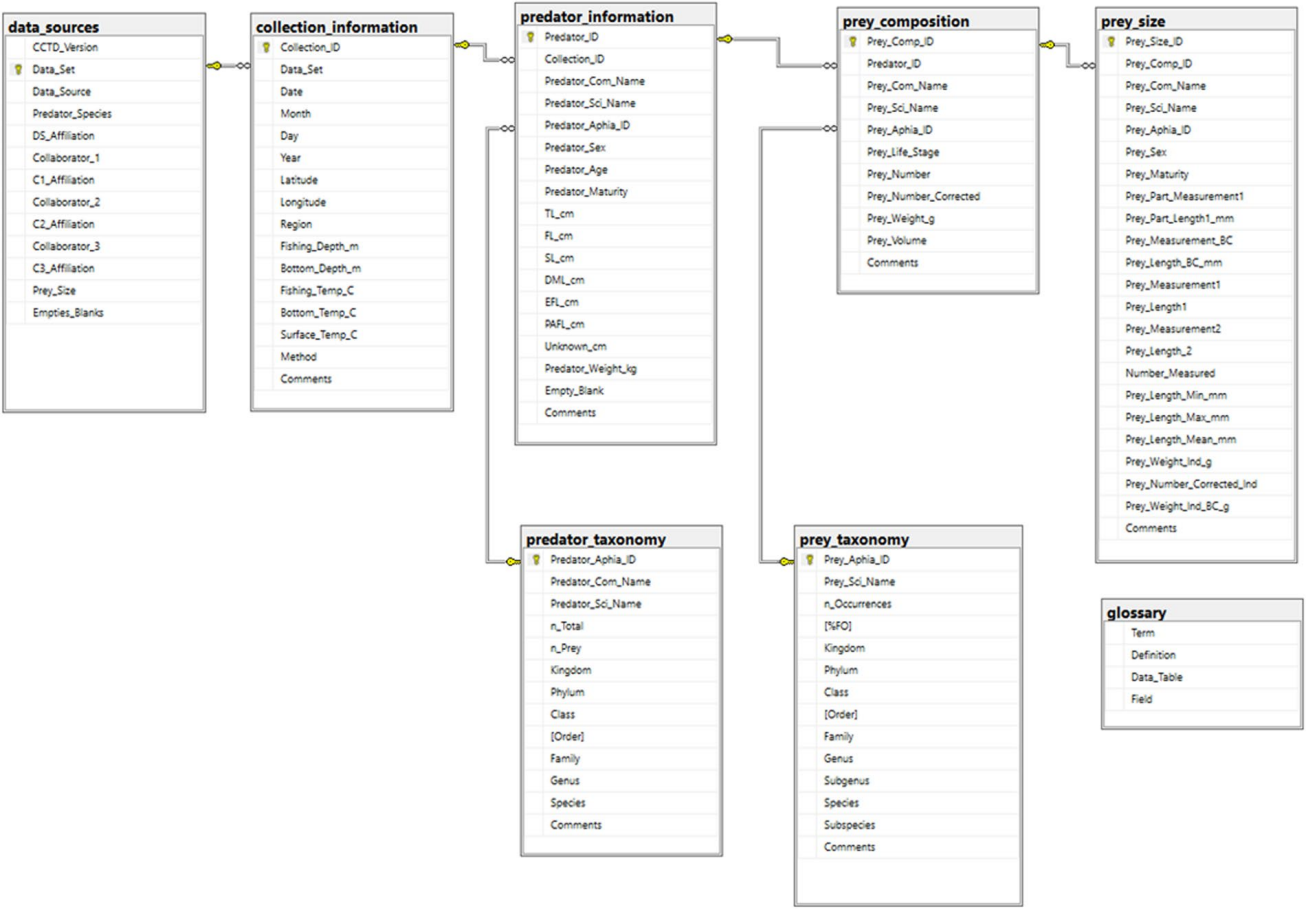
California Current Trophic Database diagram. Primary keys within tables (gold keys) and foreign key relationships between tables (infinity symbols) are indicated. Primary key = a unique identifier for each row in a table. Foreign key = a set of attributes in a field of one table that refers to the primary key field of another table. Relationships are therefore hierarchical in the direction of one (primary key, parent table) to many (foreign key, child table). TL = total length, FL = fork length, SL = standard length, DML = dorsal mantle length, EFL = eye fork length, PAFL = pre-anal fin length, BC = back-calculated, Ind = individual.

Because trophic data were derived from many sources, considerable effort was devoted to standardization. A diverse array of collection methods was used among the contributed data sets. The type and amount of relevant information also varied among studies. The hierarchical framework of a relational database, consisting of Data Sources, Collection Information, Predator Information, Prey Composition, and Prey Size tables, was developed using SQL Server Management Studio to guide data gathering and organization efforts (Fig. 1). Data sets were reviewed soon after submission and questions were communicated to the data contributor, so that each table and field was properly interpreted. Communication was maintained with data contributors while data were processed and synthesized using Microsoft Excel and R Statistical Software. This step consisted of transferring the content of each obtained data set into the relevant fields among blank versions of the specified CCTD tables. A direct link between collection information, predator information, and prey composition was required to incorporate each data set into the indicated hierarchical format. Prey size data were obtained when available, but were not required for inclusion. Final versions of each data set, reorganized among CCTD tables, were then returned to contributors for review and approval before being included in the CCTD. After several data sets were compiled, fields and field definitions were created for each CCTD table and used to identify and organize observed variability in data types and formats (Fig. 1). This effort also helped to guide additional data collection, processing, and synthesis efforts. A metadata document that explains the general content of each table and provides definitions for each associated field was created and provided for users of the CCTD as explained below (see Database Framework and Formats).

Data inputs for each field and their units were standardized for each data set before inclusion in the CCTD. Diet composition metrics consisted of either numerical or gravimetric (i.e., weight or volume) estimates. Original counts from stomach or scat samples were consistently recorded among studies and were aggregated, whereas back calculated or adjusted totals were represented separately. All weight estimates consisted of wet weight, which was standardized to grams. Volumetric estimates, often taken at sea, were presented as percentages (rather than proportions). Non-biological objects (e.g., sand, rocks, plastic) and any identified parasites were removed from diet composition data. Length estimates of predators (cm) and prey (mm) were reported among widely applicable, commonly used measurements (e.g., total length, carapace width) whenever possible; however, when a measurement from a particular study varied somewhat from standard practices or was unique, it was retained (e.g., eye to fork length for swordfish, pre-anal fin length for grenadiers). Data often were provided at unrealistic levels of precision (i.e., “false precision”) due to conversions or other calculations and were therefore rounded to values that could be substantiated for each type of measurement.

## Data Records

### Database framework and formats

The tables that constitute the CCTD and a static file of its combined contents are available at Marine Data Archive^21^, which requires users to register for a cost-free account before accessing data. The CCTD was developed using the Structured Query Language (SQL) and consists of eight data tables (Fig. 1). In addition to the five hierarchical (parent-child) tables that previously were described, two taxonomic tables, one for predators (n = 143) and one for prey (n = 1659), are included and linked to the Predator Information and Prey Composition tables (Fig. 1). A stand-alone glossary of terms also is included in the CCTD to ensure transparency and interpretation. Primary keys (unique identifier field for each row in a table) and foreign key (attributes in a child table that link back to the primary key field of a parent table) relationships are visualized in the SQL database diagram (Fig. 1).

In addition to the individual tables and static file deposited at Marine Data Archive, relational CCTD databases are available through SWFSC’s data portal. The CCTD was created in SQL and is publicly available in that format (https://oceanview.pfeg.noaa.gov/cctd/). It also is provided through SWFSC’s Environmental Research Division’s ERDDAP site^22^ (https://oceanview.pfeg.noaa.gov/erddap/search/index.html?&searchFor=SWFSC-CCTD). ERDDAP is a data server that provides a simple, consistent way to browse and download subsets of scientific data in common file formats. The CCTD is served by ERDDAP as flat tables, referred to as a “tabledap” dataset. The documentation for “tabledap” (https://coastwatch.pfeg.noaa.gov/erddap/tabledap/documentation.html) contains descriptions of the types of files available for download and provides instructions on how to download data directly in various tools and programming languages such as python, MatLab, ArcGIS, and R, which has support packages{rerddap} to facilitate easy data access. ERDDAP provides the ability to make database-like queries of the CCTD data which can include qualifiers such as “distinct()” and “orderBy()”. Every query is represented by a unique ERDDAP URL that contains all aspects of the query and can then be scripted (for example, by looping through time, location, or species) to bypass the user interface completely. Primary keys and foreign key relationships that were established in the SQL database remain the structure of the database in ERDDAP.

### Overall content

**Fig. 2:**
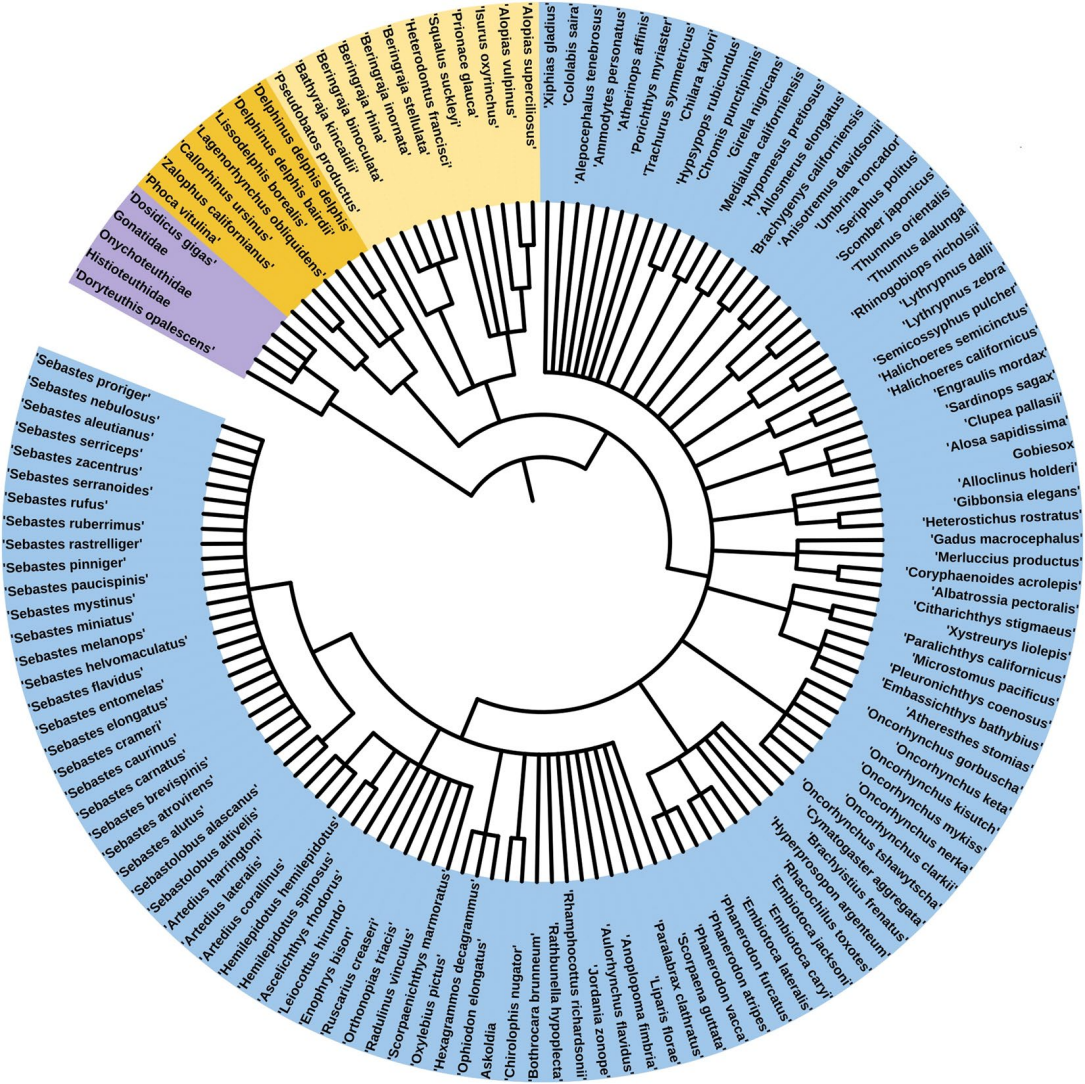
Phylogeny of marine predators included in the California Current Trophic Database, as constructed using the World Register of Marine Species (https://www.marinespecies.org/) for taxonomic designations and the Interactive Tree of Life (https://itol.embl.de/) for the construction and display of the phylogenetic tree. Taxa are arranged according to their evolutionary relationships, and organized by taxonomic classification. Purple = squids (n = 5), orange = mammals (n = 7), yellow = elasmobranch fishes (n = 12), blue = teleost fishes (n = 115). Higher taxonomic (i.e., Selachii) and generic (i.e., *Liparis*, *Artedius*, *Sebastes*) designations that include identified species (e.g., *Prionace glauca*, *Sebastes rufus*) were not included.

Food habits data were obtained from 105,694 individual predators comprising 143 taxa collected throughout the greater CCLME from 1967–2019. Predator taxa consist of squids (n = 5), elasmobranchs (n = 13), bony fishes (n = 118), and marine mammals (n = 7) (Fig. 2), with taxonomic classification information provided in the Predator Taxonomy table of the SQL database. Among these individuals, 92,430 (87.5%) had either stomach samples or scat samples containing prey whereas samples from 13,264 individuals were either empty or blank, respectively (Supplemental Table 1). These data are included in the SQL database between the Predator Information table, which contains information about the species, sex, age, maturity, size (length, weight), and prey contents (yes, no) of each individual predator, and the Prey Composition table, which includes the level of taxonomic identification, hierarchical taxonomy, and number, weight, and volume of each identified taxon in each predator’s diet. There are 227,605 records in the Prey Composition table, representing the aggregated diet composition estimates of each individual predator, and 1659 distinct prey types listed in the Prey Taxonomy table, which includes taxonomic classifications and generalized prey designations (e.g., algae, gelatinous zooplankton). A subset of the prey items included in the Prey Composition table were measured and included in the SQL Prey Size table (262,443 records), which provides information about the size and weight of individual prey items in predator diets, and the size measurement(s) taken. Since the flat data file provided on the ERDDAP site was derived from the SQL relational database, information on data records provided for the SQL database and ERDDAP flat file are consistent.

### Spatial and temporal coverage

**Fig. 3:**
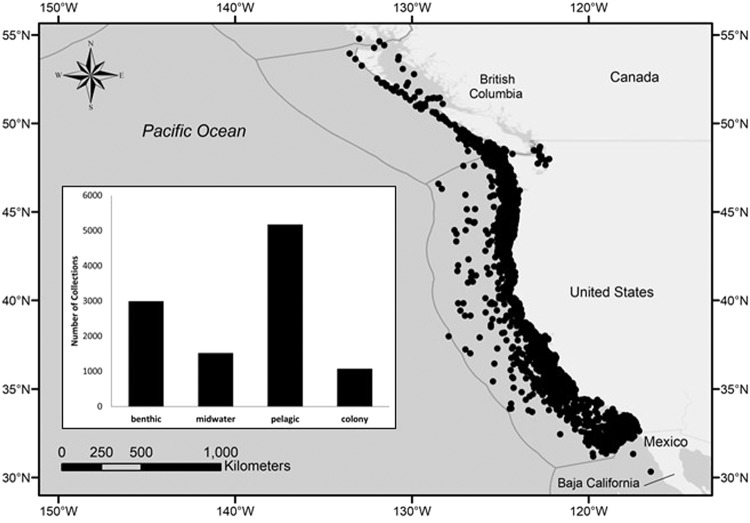
Geographic distribution of stomach and scat sample collections with latitude and longitude information (n = 9406 of 10790 total collections, or 87.2%) in the eastern North Pacific, and focused in the California Current region, between British Columbia, Canada, and Baja California, Mexico. Grey lines represent national Exclusive Economic Zone boundaries. Inset map depicts relative amount of oceanic (benthic, midwater, pelagic) and land-based (pinniped) colony collections (n = 10756).

The spatial extent of our study region ranges from Baja California (30.25° N) to Southeast Alaska (54.77° N), from nearshore waters to beyond the EEZ, and includes samples collected from the surface to the benthos (Fig. 3). The great majority of samples were collected within the region extending from Vancouver Island to Baja California, which encompasses all but the southernmost portion of the CCLME^23^. We established standard criteria for defining a collection event among studies, including samples collected on the same day, with consistent gear, and at the same location and depth. Through this process, all individual predators were assigned to one of 10,790 collections contained in the SQL Collection Information table. Geographic coordinates were provided for most collections (n = 9406, or 87.2%), and all but 8 collections could at least be associated with a general region (e.g., Southern California, Central California, Pacific Northwest). We divided collections within the water column to include three regions: pelagic (<~30 m from the surface), benthic (<~30 m from the benthos), and midwater (open water between pelagic and benthic regions). Nearly half of the collections were from pelagic habitats (48.2%, n = 5173) with benthic habitats also well represented (27.8%, n = 2992). By contrast, midwater collections and land-based (pinniped colony) collections were fewer (Fig. 3), although the duration of sampling and the overall sample size for the pinniped colony collections in the Southern California Bight were quite robust.

Samples are included from collections made during 1967–2019, but most were acquired during the last two decades. Only 2.4% of samples (n = 292) with temporal information were collected prior to 1980. By contrast, 62.1% (n = 6698) of collections occurred after 1999. The number of collections provided for the 1980s (18.2%, n = 1961) and 1990s (17.3%, n = 1867) was similar. It should be noted that the number of collections is not a reliable indicator of the number of individual stomach or scat samples obtained in a collection. For example, some gear, such as hook and line or spearing collections, takes a single individual in a collection event whereas others (e.g., trawl, longline) collect many.

### Predator and prey coverage

Among predator taxa, bony fishes contributed the greatest proportion of samples containing prey (70.2%, n = 64,903); however, marine mammals, which were far less speciose, also provided a substantial proportion of these samples (25.8%, n = 23,820). Furthermore, more than half of the bony fish samples with prey contents were contributed by two species, Pacific Hake (n = 19,244) and Chinook Salmon (n = 15,973), and two-thirds of bony fish taxa had < 100 samples with prey contents (n = 66.1%, n = 78). Marine mammal samples were similarly dominated by the relative contribution of California Sea Lions (n = 20,197) and, to a lesser extent, Harbor Seals (n = 2883). Despite their large sizes and difficulty of capture and handling, chondrichthyan taxa, comprising sharks, skates, and rays, were relatively well sampled, with > 100 stomach samples with prey contents available for 9 of 13 taxa (Supplemental Table 1). A substantial amount of stomach samples containing prey were provided for Humboldt Squid (n = 1136) but most other cephalopod taxa, consisting exclusively of squids, were represented by relatively few samples and family-level identifications (Supplemental Table 1). When predators are aggregated by management group or habitat, 31.9% (n = 29,522) of samples were derived from pelagic taxa, including salmonids (n = 24.4%, n = 22,554), highly migratory species (3.0%, n = 2773), and other pelagic species (n = 4.5%, n = 4195). Groundfishes and demersal, nearshore fishes also are well-represented (42.3%, n = 39,088). Extensive time series are available for some predators (e.g., California Sea Lion, Pacific Hake, Chinook Salmon), with widespread geographic coverage exhibited for Chinook Salmon and some groundfishes (Pacific Hake, Dover Sole, Sablefish, Lingcod).

Prey taxa in the CCTD included five kingdoms and at least 25 phyla; however, the relative amount of representative prey from all kingdoms except Animalia was trivial (Table 2). Among animal phyla, chordate and arthropod prey were the main dietary items, with taxa from each group occurring in nearly 60% of stomach or scat samples (Table 2). Chordate prey was overwhelmingly composed of bony fishes of the Class Actinopterygii (%Frequency of Occurrence, %FO = 50.6) whereas arthropod prey mostly consisted of malacostracan crustaceans (%FO = 47.9). Molluscan prey, mostly represented by Cephalopods (%FO = 19.1), was present in about one-fourth of all predator samples (Table 2). The importance of euphausiids to the predators in the CCLME was noteworthy (Order Euphausiacea, %FO = 26.3), and this prey group occurred in a higher portion of samples than amphipods (Order Amphipoda, %FO = 11.7%) or decapods (Order Decapoda, %FO = 17.5). Clupeiforms (%FO = 17.3) and scopaeniforms (%FO = 13.3%) were the most commonly ingested fishes, whereas Myopsida (%FO = 13.0%) was the most frequently consumed cephalopod class.

**Table 2:** Prey composition summarized for all predators in the California Current Trophic Database by kingdom and phylum.

Kingdom	Phylum	n_occurrences_	%FO
Protista	unidentified	24	0.03
Protozoa	Sarcomastigophora	1	0.00
Chromista	Foraminifera	120	0.13
	Cercozoa	233	0.25
	Ochrophyta	1,078	1.17
	Myzoza	3	0.00
	unidentified	8	0.01
Plantae	Rhodophyta	716	0.77
	Chlorophyta	65	0.07
	Tracheophyta	129	0.14
	unidentified	520	0.56
Animalia	Porifera	58	0.06
	Ctenophora	173	0.19
	Cnidaria	1,327	1.44
	Chordata	53,471	57.86
	Echinodermata	2,576	2.79
	Arthropoda	55,125	59.65
	Nematoda	121	0.13
	Chaetognatha	242	0.26
	Platyhelminthes	45	0.05
	Annelida	5,088	5.51
	Sipuncula	156	0.17
	Mollusca	21,966	23.77
	Brachiopoda	3	0.00
	Phoronida	4	0.00
	Bryozoa	673	0.73
	Entoprocta	2	0.00
	Nemertea	76	0.08
	unidentified	8,243	8.92

n_occurrences_ = number of stomach or scat samples that contained a prey taxon. %FO = percentage frequency of occurrence among all predators that had taxonomically identified prey contents (n = 92,418).

## Technical Validation

All data for each data set were standardized among the various tables in the CCTD according to the definitions and criteria established for each field. Taxonomic nomenclature and resolution varied among contributed data sets, necessitating the development of standard practices to ensure reliable comparisons. The World Register of Marine Species (WoRMS) (https://www.marinespecies.org/) was used for taxonomic reference, and scientific names from each data set were updated accordingly. Additional taxonomic resources (e.g., recent primary literature, California Academy of Fishes Fish Catalog) were incorporated when WoRMS designations were dated or unsubstantiated. All predators were identified taxonomically. Polypheletic or non-taxonomic categories, however, were sometimes used for prey contents (e.g., gelatinous zooplankton, phytoplankton, detritus) when taxonomic designations were not provided. Each sample collection event with geographic coordinates was plotted to identify errors, and all dubious coordinate information was removed from collections. Length and weight estimates for predators and prey were checked against maximum reported estimates to screen for any obvious errors. Samples that were missing numerical or gravimetric estimates for a particular prey taxon were retained in the database to enable their use for occurrence estimates.

## Usage Notes

Before accessing the CCTD, interested users are encouraged to consult the primary literature associated with each desired data set to understand details regarding collection, processing, and identification methods and how they vary among studies (Table 1). This step is especially important because unknown differences among studies can confound objectives or bias results. For example, though the method of data collection is provided in the CCTD and may be generally consistent among certain data sets, gear size and diel differences in data collection must be obtained from the primary source(s) of the desired data set(s). A User Guide and metadata are available online to provide additional guidance and reference to interested users (https://oceanview.pfeg.noaa.gov/cctd/). Definitions for every field in each data table are provided in the metadata document to help the user understand the contents of the CCTD.

Caveats and considerations are provided for some species to address recent ambiguities in their taxonomic status. As an example, Blue Rockfish (*Sebastes mystinus*) predator and prey samples undoubtedly contain some portion of the formerly cryptic Deacon Rockfish (*S. diaconus*). This is especially true of samples collected north of Monterey Bay^24^. The taxonomic status of Short-Beaked (*Delphinus delphis delphis*) and Long-Beaked (*D. delphis bairdii*) Common Dolphins is in flux, but WoRMS considers both subspecies to be valid. We retained this distinction at the subspecies level for both predators in the CCTD. The Sandpaper Skate, *Bathyraja kincaidii*, was considered a synonym of the Bering Skate, *B. interrupta*, until recently; however, it is now understood that 1) *B. kincaidii* is a valid species, and that 2) *B. kincaidii* replaces *B. interrupta* in the CCLME, where it is included in the CCTD as a predator and prey species^25,26^.

One of the main goals of developing the CCTD was to facilitate the advancement of trophic-based research to support fisheries, protected species, and ecosystem-based marine resource management objectives in the CCLME. To that end, several projects that leverage the database have been initiated, and some are nearing completion. These include a comparative analysis of Chinook Salmon and Pacific Hake trophic biogeography^27^, an Atlantis Ecosystem Model update, salmon life-cycle modeling, investigations of cannibalism and other trophic linkages for a Pacific Hake bioenergetic model, modeling California sea lion prey preference and functional response to inform ecosystem modeling, developing a Model of Intermediate Complexity for Ecosystem assessment (MICE) to forecast sardine abundance by parameterizing natural (predation) mortality, and the determination of trophic guilds for a diverse array of marine predators.

We hope that researchers within the CCLME will not only use the CCTD to obtain data but will be encouraged to share their data from contemporary or historical trophic studies to facilitate the growth of this database and increase its value. To this end, a few data sets that were being compiled during the initial data mining effort are now complete and available, and some additional sources were located after the content of the CCTD was finalized. SWFSC is supportive of the maintenance and growth of the CCTD, and has provided financial support to initiate a new phase of data mining to acquire these additional data sets and any others that can be located. Our initial data mining effort was focused within NMFS, but we plan to expand our search to include more academic researchers and graduate students and museum archives, with new data incorporated an updated version made publicly available after all newly identified data sets are obtained. Although the structure and format of the CCTD (e.g., data tables and their relationships) and the platforms that currently serve it will be maintained, the content will be augmented with additional diet composition and (when available) prey size data. It is therefore important to note that this Data Descriptor was peer reviewed in 2023 based on the data that were available in the initial version of the CCTD and that details regarding content and data records will not apply to the planned update and any future versions.

## Supplementary information

Supplemental Information

## Data Availability

No custom code was created in the generation or processing of data sets. R version 3.6.0, Notepad version 7.7.1, and Microsoft Excel 2016 were used to process each submitted data set and to build the eight previously described data tables. SQL Server Management Studio 18.12.1 was used to establish primary and foreign key relationships between these data tables to create the CCTD.
