# Supplementary material for: A multi-predator trophic database for the California Current Large Marine Ecosystem
Source: Sci Data. 2023 Jul 27;10:496. doi: 10.1038/s41597-023-02399-2 (PMC10374555; doi:10.1038/s41597-023-02399-2)
Supplement: Supplementary file 1 — Supplemental Information [file 41597_2023_2399_MOESM1_ESM.pdf]

Table of Contents

Supplemental Methods – Page 1

Table S1 – Pages 2 and 3

Supplemental Methods

Each of the obtained data sets had at least one associated publication that describes collection and processing methods, as indicated in Table 1. However, one data set (Data Set 20 – Slope Groundfishes) was compiled from several distinct survey cruises and is only partially documented by the supporting literature (Laidig et al. 1997). Details concerning this data set are therefore provided here.

Fishes were collected using bottom trawl gear on nine National Marine Fisheries Service research cruises extending from northern Oregon (45.211° N) to southern California (33.427° N) during September-April of 1987-1992, with the majority of the hauls conducted during 1988-1990 (n = 313 of 344). Six species were primarily sampled for diet composition at depths ranging from 104-1421 m (median = 644 m): Giant Grenadier (*Albatrossia pectoralis*, n = 162), Shortspine Thornyhead (*Sebastolobus alascanus*, n = 1590), Longspine Thornyhead (*S. altivelis*, n = 1419), Sablefish (*Anoplopoma fimbria*, n = 2022), Dover Sole (*Microstomus pacificus*, n = 1638), and Deep-Sea Sole (*Embassicthys bathybius*, n = 374). Fishes of these species were identified and up to 15 individuals were sampled per haul. Selection of individuals was based initially on size (the largest and smallest individuals were prioritized), and the remaining individuals were chosen haphazardly. Trivial numbers of three additional species were sampled: California Slickhead (*Alepocephalus tenebrosus*, n = 3), Twoline Eelpout (*Bothrocara brunneum*, n = 4), and Arrowtooth Flounder (*Atheresthes stomias*, n = 1). All sampled fishes were measured to the nearest cm (either standard length or pre-anal (fin) length, for grenadiers), and their stomachs were excised and fixed in a 10% formalin solution. In the laboratory, stomachs were removed from formalin and rinsed in freshwater before processing. Stomach contents were identified to the lowest possible taxa and enumerated, and the total length of freshly dead (i.e., slight degradation) prey items was measured along the longest body axis.

Class	Order	Family	Common Name	Scientific Name	n_{total}	n_{prey}	Data Source(s)
Cephalopoda	Myopsida	Loliginidae	Market Squid	*Doryteuthis opalescens*	226	119	8
	Oegopsida	Histioteuthidae	Cock-Eyed Squid	Histioteuthidae	53	47	1
		Ommastrephidae	Humboldt Squid	*Dosidicus gigas*	1304	1136	15
		Gonatidae	Armhook Squid	Gonatidae	4	4	1
		Onychoteuthidae	Hooked Squid	Onychoteuthidae	8	8	1
Chondrichthyes			Shark	Selachii	1	1	10
	Heterodontiformes	Heterodontidae	Horn Shark	*Heterodontus francisci*	4	4	19
	Lamniformes	Alopidae	Bigeye Thresher Shark	*Alopias superciliosus*	51	45	24
			Common Thresher Shark	*Alopias vulpinus*	610	432	24
		Lamnidae	Shortfin Mako Shark	*Isurus oxyrinchus*	501	366	24
	Carcharhinformes	Carcharhinidae	Blue Shark	*Prionace glauca*	228	167	8, 10, 24
	Squaliformes	Squalidae	Pacific Spiny Dogfish	*Squalus suckleyi*	206	120	8, 10
	Rajiformes	Rhinobatidae	Shovelnose Guitarfish	*Pseudobatos productus*	5	5	19
		Arhynchobatidae	Sandpaper Skate	*Bathyraja kincaidii*	125	125	4
		Rajidae	Big Skate	*Beringraja binoculata*	211	211	4
			California Skate	*Beringraja inornata*	264	263	4
			Longnose Skate	*Beringraja rhina*	544	544	4
			Starry Skate	*Beringraja stellulata*	110	110	4
Actinopterygii		Engraulidae	Northern Anchovy	*Engraulis mordax*	232	222	7, 8, 10
		Clupeidae	Pacific Herring	*Clupea pallasii*	503	450	7, 8, 10
			American Shad	*Alosa sapidissima*	8	8	7, 10
			Pacific Sardine	*Sardinops sagax*	327	181	7, 8, 10
	Argentiniformes	Alepocephalidae	California Slickhead	*Alepocephalus tenebrosus*	3	3	20
	Osmeriformes	Osmeridae	Whitebait Smelt	*Allosmerus elongatus*	301	186	7, 8, 19
			Surf Smelt	*Hypomesus pretiosus*	500	406	7, 8
	Salmoniformes	Salmonidae	Cutthroat Trout	*Oncorhynchus clarkii*	12	11	8
			Pink Salmon	*Oncorhynchus gorbuscha*	21	21	13, 17
			Chum Salmon	*Oncorhynchus keta*	166	161	8, 13, 17
			Coho Salmon	*Oncorhynchus kisutch*	5613	5318	8, 9, 10, 14, 17
			Steelhead	*Oncorhynchus mykiss*	1072	1022	8, 9, 17
			Sockeye Salmon	*Oncorhynchus nerka*	49	48	9
			Chinook Salmon	*Oncorhynchus tshawytscha*	18547	15973	8, 9, 10, 14, 16, 17
	Gadiformes	Merlucciidae	Pacific Hake	*Merluccius productus*	23409	19244	1, 3, 8, 10
		Macrouridae	Giant Grenadier	*Albatrossia pectoralis*	300	205	1, 20
			Pacific Grenadier	*Coryphaenoides acrolepis*	40	33	1
		Gadidae	Pacific Cod	*Gadus macrocephalus*	1	1	10
	Ophidiiformes	Ophidiidae	Spotted Cusk-Eel	*Chilara taylori*	30	26	19
	Batrachoidiformes	Batrachoididae	Specklefin Midshipman	*Porichthys myriaster*	1	1	19
	Atheriniformes	Atherinidae	Topsmelt Silverside	*Atherinops affinis*	40	26	19
	Beloniformes	Scomberesocidae	Pacific Saury	*Cololabis saira*	194	158	8, 18
	Gobiesociformes	Gobiesocidae	Clingfishes	*Gobiesox*	2	2	18
	Gasterosteiformes	Aulorhynchidae	Tubesnout	*Aulorhynchus flavidus*	9	9	18
	Scorpaeniformes	Anoplopomatidae	Sablefish	*Anoplopoma fimbria*	2860	2619	1, 7, 8, 20
		Cottidae	Sculpin	*Artedius*	1	1	18
			Coralline Sculpin	*Artedius corallinus*	20	17	18, 19
			Scalyhead Sculpin	*Artedius harringtoni*	124	124	18
			Smoothhead Sculpin	*Artedius lateralis*	4	1	18
			Rosylip Sculpin	*Ascelichthys rhodorus*	2	2	18
			Buffalo Sculpin	*Enophrys bison*	3	3	18
			Red Irish Lord	*Hemilepidotus hemilepidotus*	1	1	18
			Brown Irish Lord	*Hemilepidotus spinosus*	2	2	18
			Longfin Sculpin	*Jordania zonope*	23	23	18
			Lavender Sculpin	*Leiocottus hirundo*	25	21	19
			Snubnose Sculpin	*Orthonopias triacis*	79	78	18, 19
			Smootgum Sculpin	*Radulinus vinculus*	3	3	18
			Roughcheek Sculpin	*Ruscarius creaseri*	9	7	19
			Cabezon	*Scorpaenichthys marmoratus*	9	8	8, 18, 19
		Rhamphocottidae	Grunt Sculpin	*Rhamphocottus richardsonii*	1	1	18
		Liparidae	Snailfish	*Liparis*	1	1	18
			Tidepool Snailfish	*Liparis florae*	1	1	18
		Hexagrammidae	Kelp Greenling	*Hexagrammos decagrammus*	436	436	18
			Lingcod	*Ophiodon elongatus*	1915	1235	2, 8, 18, 19
			Painted Greenling	*Oxylebius pictus*	72	72	18, 19
		Scorpaenidae	California Scorpionfish	*Scorpaena guttata*	28	18	19
		Sebastidae	Rockfish	*Sebastes*	77	23	8, 18
			Rougheye Rockfish	*Sebastes aleutianus*	27	20	1
			Pacific Ocean Perch	*Sebastes alutus*	199	133	1
			Kelp Rockfish	*Sebastes atrovirens*	45	32	19
			Silvergray Rockfish	*Sebastes brevispinis*	43	25	1
			Gopher Rockfish	*Sebastes carnatus*	1017	736	21
			Copper Rockfish	*Sebastes caurinus*	1	1	18
			Darkblotched Rockfish	Sebastes crameri	30	22	1, 8
			Greenstriped Rockfish	*Sebastes elongatus*	56	47	1

Class	Order	Family	Common Name	Scientific Name	n_{total}	n_{prey}	Data Source(s)
			Widow Rockfish	*Sebastes entomelas*	59	59	1, 8
			Yellowtail Rockfish	*Sebastes flavidus*	504	451	1, 5, 8, 10, 18
			Rosethorn Rockfish	*Sebastes helvomaculatus*	9	8	1
			Black Rockfish	*Sebastes melanops*	285	193	1, 8, 18
			Vermilion Rockfish	*Sebastes miniatus*	1	1	18
			Blue Rockfish	*Sebastes mystinus*	2369	1986	18, 19
			China Rockfish	*Sebastes nebulousus*	6	5	18
			Bocaccio	*Sebastes paucispinis*	9	5	1, 19
			Canary Rockfish	*Sebastes pinniger*	90	69	1, 8, 19
			Redstripe Rockfish	*Sebastes proriger*	28	24	1
			Grass Rockfish	*Sebastes rastrelliger*	4	2	19
			Yelloweye Rockfish	*Sebastes ruberriums*	1	1	1
			Bank Rockfish	*Sebastes rufus*	9	9	8
			Olive Rockfish	*Sebastes serranoides*	250	152	19
			Treefish	*Sebastes serriceps*	29	15	19
			Sharpchin Rockfish	*Sebastes zacentrus*	21	8	1
			Shortspine Thornyhead	*Sebastolobus alascanus*	2257	2082	1, 20
			Longspine Thornyhead	*Sebastolobus altivelis*	1747	1698	1, 20
	Perciformes	Carangidae	Pacific Jack Mackerel	*Trachurus symmetricus*	2604	1387	7, 8, 10, 19
		Serranidae	Kelp Bass	*Paralabrax clathratus*	151	111	19
		Haemulidae	Xantic Sargo	*Anisotremus davidsonii*	11	7	19
			California Salema	*Xenistius californiensis*	20	13	19
			Queen Croaker	*Seriphus politus*	37	34	19
			Yellowfin Drum	*Umbrina roncador*	28	22	19
		Kyphosidae	Opaleye	*Girella nigricans*	14	13	19
			Halfmoon	*Medialuna californiensis*	15	15	19
		Embiotocidae	Kelp Perch	*Brachyistius frenatus*	185	149	19
			Shiner Perch	*Cymatogaster aggregata*	126	84	19
			Black Surfperch	*Embiotoca jacksoni*	135	111	19
			Striped Surfperch	*Embiotoca lateralis*	170	167	18
			Walleye Surfperch	*Hyperprosopon argenteum*	62	51	19
			Rainbow Seaperch	*Hypsurus caryi*	9	9	18
			Sharpnose Seaperch	*Phanerodon atripes*	7	6	18
			White Seaperch	*Phanerodon furcatus*	11	9	19
			Rubberlip Seaperch	*Rhacochilus toxotes*	12	8	19
			Pile Perch	*Phanerodon vacca*	40	25	19
		Pomacentridae	Blacksmith	*Chromis punctipinnis*	134	121	19
			Garibaldi	*Hypsypops rubicundus*	66	65	19
		Labridae	Rock Wrasse	*Halichoeres semicinctus*	212	210	19
			Senorita	*Halichoeres californicus*	212	208	18, 19
			California Sheephead	*Semicossyphus pulcher*	177	175	19
		Zoarcidae	Twoline Eelpout	*Bothrocara brunneum*	4	4	20
		Bathymasteridae	Stripefin Ronquil	*Rathbunella hypoplecta*	1	1	19
		Stichaeidae	Prickleback	*Askoldia*	1	1	18
			Mosshead Warbonnet	*Chirolophis nugator*	1	1	18
		Ammodytidae	Pacific Sandlance	*Ammodytes personatus*	77	68	8
		Clinidae	Spotted Kelpfish	*Gibbonsia elegans*	19	19	19
			Giant Kelpfish	*Heterostichus rostratus*	11	7	19
		Labrisomidae	Island Kelpfish	*Alloclinus holderi*	22	22	19
		Gobiidae	Bluebanded Goby	*Lythrypnus dalli*	78	77	19
			Zebra Goby	*Lythrypnus zebra*	29	29	19
			Blackeye Goby	*Rhinogobiops nicholsii*	98	97	18, 19
		Scombridae	Pacific Mackerel	*Scomber japonicus*	659	440	7, 8, 10
			Albacore	*Thunnus alalunga*	913	750	11
			Pacific Bluefin Tuna	*Thunnus orientalis*	963	721	12
		Xiphiidae	Broadbill Swordfish	*Xiphias gladius*	299	292	24
	Pleuronectiformes	Pleuronectidae	Arrowtooth Flounder	*Atheresthes stomias*	381	190	1, 20
			Deepsea Sole	*Embassichthys bathybius*	545	504	1, 20
			Dover Sole	*Microstomus pacificus*	2484	2408	1, 20
			C-O Sole	*Pleuronichthys coenosus*	51	46	19
		Paralichthyidae	Speckled Sanddab	*Citharichthys stigmaeus*	27	22	18, 19
			California Flounder	*Paralichthys californicus*	12	7	19
			Fantail Flounder	*Xystreurys liolepis*	1	1	19
Mammalia	Artiodactyla	Delphinidae	Long-Beaked Common Dolphin	*Delphinus delphis bairdii*	49	49	24
			Short-Beaked Common Dolphin	*Delphinus delphis delphis*	259	259	24
			Pacific White-Sided Dolphin	*Lagenorhynchus obliquidens*	25	25	24
	Carnivora	Phocidae	Northern Right Whale Dolphin	*Lissodelphis borealis*	56	56	24
			Harbor Seal	*Phoca vitulina*	2947	2883	22, 23
		Otariidae	Northern Fur Seal	*Callorhinus ursinus*	351	351	22
			California Sea Lion	*Zalophus californianus*	20266	20197	6, 22

Table S1. Phylogeny, sample sizes, and data source(s) for predator taxa in the California Current Trophic Database. Sample size information includes empty stomachs or blank scat samples (n_{total}) and those with prey contents (n_{prey}), when available. Detailed information for data source numbers is provided in Table 1.
